# How storey configuration and number of transition points shape opportunities for outdoor access in residential care facilities: implications for age-friendly environments

**DOI:** 10.1186/s12877-026-08094-2

**Published:** 2026-08-12

**Authors:** Madeleine Liljegren, Anna Bengtsson, Susanna Nordin, Göran Lindahl, Helle Wijk

**Affiliations:** 1 Institute of Health and Care Sciences, University of Gothenburg, Arvid Wallgrens Backe, House 1, Box 457, Gothenburg, 405 30 Sweden; 2 Centre for a DEMentia-Friendly Society With A Person-Centred Mindset (DEMSAM), Institute of Health and Care Sciences, University of Gothenburg, Arvid Wallgrens Backe, House 1, Box 457, Gothenburg, 405 30 Sweden; 3 Department of Architecture and Civil Engineering, Chalmers University of Technology, Sven Hultins Gata 6, Gothenburg, 412 96 Sweden; 4 Department of People and Society, The Swedish University of Agricultural Sciences, Slottsvägen 5, Alnarp, 234 56 Sweden; 5 School of Health and Welfare, Dalarna University, Högskolegatan 2, Falun, 791 88 Sweden

**Keywords:** Architectural design, Multi-methods design, Nursing home, Older people, Outdoor environment, Person-centred practice, Storey configuration

## Abstract

**Background:**

Age-friendly environments are increasingly recognised as essential for supporting participation, autonomy, and healthy ageing. In residential care facilities (RCFs) for older adults, the built environment interacts with organisational practices to shape opportunities for person-centred care and rehabilitation. However, limited knowledge exists regarding how architectural configurations influence access to immediate outdoor environments.

**Aim:**

To examine how storey configuration (single- versus multi-storey) and number of transition points were associated with opportunities for immediate access to outdoor environments within and adjacent to RCFs.

**Methods:**

A multi-methods design was used, comprising national mapping of storey configuration of 1,603 Swedish RCFs, mapping of transition points at three RCFs, and walking interviews with twelve older adults and eleven care workers.

**Results:**

Single-storey RCFs involved fewer transition points between indoor and outdoor environments, shaping opportunities for immediate outdoor access. In contrast, multi-storey RCFs required additional transitions, including corridors, elevators, and stairs, which increased dependence on care workers and constrained opportunities for outdoor engagement. Architectural conditions interacted with organisational routines, shaping older adults' opportunities for outdoor access and the practical possibilities for outdoor person-centred care and rehabilitation.

**Conclusions:**

Storey configuration and number of transition points constitute structural conditions that shape outdoor access in RCFs. These findings have implications for age-friendly design and planning of RCFs as well as for person-centred care and rehabilitation.

## Background

Population ageing is increasing the demand for residential care facilities (RCFs) as long-term living environments for older adults with complex care and rehabilitation needs. As frail older adults spend the final years of life in RCFs, these settings have become important components of age-friendly environments. Age-friendly environments refer to physical and social environments that enable older adults to age actively by supporting participation, autonomy, health, and well-being across different contexts of everyday life [18, 51, 52]. Increasingly, age-friendly environments are also concerned with equity and inclusion, recognising that environmental conditions should enable participation among older adults with diverse functional abilities [10, 52]. Contemporary research emphasises that supportive environments emerge through interactions between physical design, organisational conditions, and everyday experiences rather than through environmental features alone [4, 35, 42]. RCFs therefore represent complex living environments where housing, care and rehabilitation practices, and spatial conditions intersect [16, 49].

While age-friendly environment research has traditionally focused on neighbourhood-level resources that support ageing in place, including housing, transportation, public spaces, and community services [7], less attention has been directed towards the immediate outdoor environments associated with RCFs. In this study, outdoor environments refer to gardens, patios, balconies, and other spaces directly connected to RCFs [27], while outdoor access refers to opportunities to reach, enter, and use these environments independently or with support from care workers. For older adults living in RCFs, such environments provide opportunities for participation, meaningful activities, and contact with nature within everyday life [13, 53].

Research from neuroarchitecture and therapeutic landscape perspectives suggests that built environments influence health and well-being through interconnected physiological, cognitive, emotional, and social processes [2, 15, 33]. Access to outdoor environments and contact with nature have been associated with improved physical function and mental well-being among older adults [1, 34, 38]. In RCFs, garden use has further been linked to improved quality of life and reduced behavioural and psychological symptoms among older adults living with dementia [47, 48]. Outdoor environments are therefore increasingly recognised as active components of care and rehabilitation rather than supplementary amenities [13, 36, 41]. Many older adults living in RCFs require care worker support to access outdoor environments, and regular outdoor stays are recommended in general [50] and as part of everyday practice within RCFs [47]. Beyond their benefits for older adults, outdoor environments may also benefit care workers by reducing work-related stress and supporting well-being [12, 30, 39]. Despite these benefits, outdoor environments remain underused in everyday care and rehabilitation within Swedish RCFs [25, 26]. Previous research has identified organisational aspects, including staffing resources, routines, workplace cultures, and older adults’ functional abilities, as important determinants of outdoor participation [25, 47]. In Sweden, there is no national regulation specifying older adults’ opportunities for outdoor access in RCFs, and only 8% of municipalities have established routines for organising outdoor care and rehabilitation [45]. Consequently, opportunities for outdoor participation depend not only on the presence of outdoor environments but also on the conditions that enable their everyday use. In Swedish RCFs, care and rehabilitation are typically delivered through multidisciplinary teams including assistant nurses, nurses, occupational therapists, and physiotherapists, with municipalities responsible for organising care and rehabilitation [44]. Understanding how architectural design contributes to these conditions, and how environmental possibilities interact with organisational practices, is therefore increasingly important [9, 19].

Although recent research has increased attention to green infrastructure surrounding RCFs and access to outdoor environments [24, 40], less is known about how the internal spatial organisation of these facilities influences older adults’ actual opportunities to access outdoor environments. One underexplored aspect of RCF design is storey configuration. In this study, storey configuration refers to whether RCFs are designed as single- or multi-storey buildings. This configuration may influence accessibility, movement patterns, and the effort required to move between indoor and outdoor environments. Single-storey RCFs may provide more direct connections to gardens and patios, whereas multi-storey RCFs often require additional movement through corridors, elevators, or stairs to reach outdoor environments. Closely related to storey configuration are transition points, referring to the physical elements and spatial passages negotiated when moving between indoor and outdoor environments, including doors, thresholds, corridors, elevators, and stairs [20]. These elements may influence the degree of independence, effort, and support required for outdoor access. Although balconies and rooftop terraces can provide alternative outdoor opportunities in multi-storey buildings, they may offer fewer possibilities for everyday activities and contact with nature compared with ground-level outdoor environments [8]. However, how number of transition points collectively influence outdoor access across different building configurations remains insufficiently explored.

In the context, limited knowledge exists regarding the distribution of single-and multi-storey RCFs, the relationship between storey configuration and number of transition points, and how these environmental conditions are experienced by older adults and care workers. Although situated within Sweden, these questions are internationally relevant, as many countries face similar challenges in developing RCFs environments that focus on accessibility, autonomy, safety, and resource constraints [52]. Understanding how architectural configurations influence outdoor access is therefore important for developing inclusive design approaches that support participation among older adults with diverse functional abilities. While previous research has examined access to outdoor environments [24], this study advances knowledge by identifying storey configuration and number of transition points as structural conditions influencing everyday outdoor access. By integrating national-level mapping, environmental analyses, and lived experiences, the study examines how architectural configurations shape opportunities for outdoor access in RCFs. This study contributes to age-friendly environment scholarship by shifting attention from the presence of outdoor environments to the architectural pathways required to reach them. By examining storey configuration and number of transition points as structural conditions for outdoor access, the study extends current understandings of how built environments shape opportunities for outdoor access in RCFs.

## Theoretical perspectives

This study is grounded in an age-friendly environments perspective, which provides a framework for understanding how physical, social, and organisational conditions interact to shape older adults’ opportunities for participation and autonomy in later life [51, 52]. Several related perspectives have informed research on ageing and the built environment. Therapeutic landscapes and place attachment emphasise how places can promote well-being, identity, and meaningful engagement [11, 15, 23], complementing an age-friendly environments perspective. More directly relevant, person–environment fit and accessibility highlight how interactions between individual capacities and environmental demands shape opportunities for participation and autonomy [22]. While these perspectives provide important contextual background, this study primarily draws on age-friendly environments, person-centred practice, and the zone model to examine how storey configurations and number of transition points shape outdoor access in RCFs.

### Person-centred practice

The study is further informed by a person-centred practice framework [31, 32], which provides an ethical foundation for examining how care and rehabilitation can be organised around older adults’ preferences, needs, and opportunities for participation. Within this perspective, person-centred practice is understood as emerging through interactions between care and rehabilitation practices, individual capacities, and environmental conditions. However, person-centred practice is often operationalised primarily through interpersonal relationships and care and rehabilitation processes, whereas the role of the physical environment as a prerequisite for exercising choice and participation has received less attention. The built environment is therefore not merely a background for care and rehabilitation but an active component that may support or hinder opportunities for choice, independence, meaningful activities, and everyday routines. In relation to outdoor access, architectural configurations may influence the extent to which person-centred care and rehabilitation can be realised in practice. For example, environments that reduce physical barriers and simplify movement between indoor and outdoor environments may facilitate spontaneous outdoor activities, whereas complex spatial arrangements may increase dependence on support from care workers. Thus, the study considers the built environment as a condition that shapes the possibilities for person-centred practice rather than as a separate aspect from care and rehabilitation delivery. Furthermore, care workers in RCFs see the potential in using outdoor environments as arenas for person-centred care and rehabilitation [25].

### The zone model

**Fig. 1:**
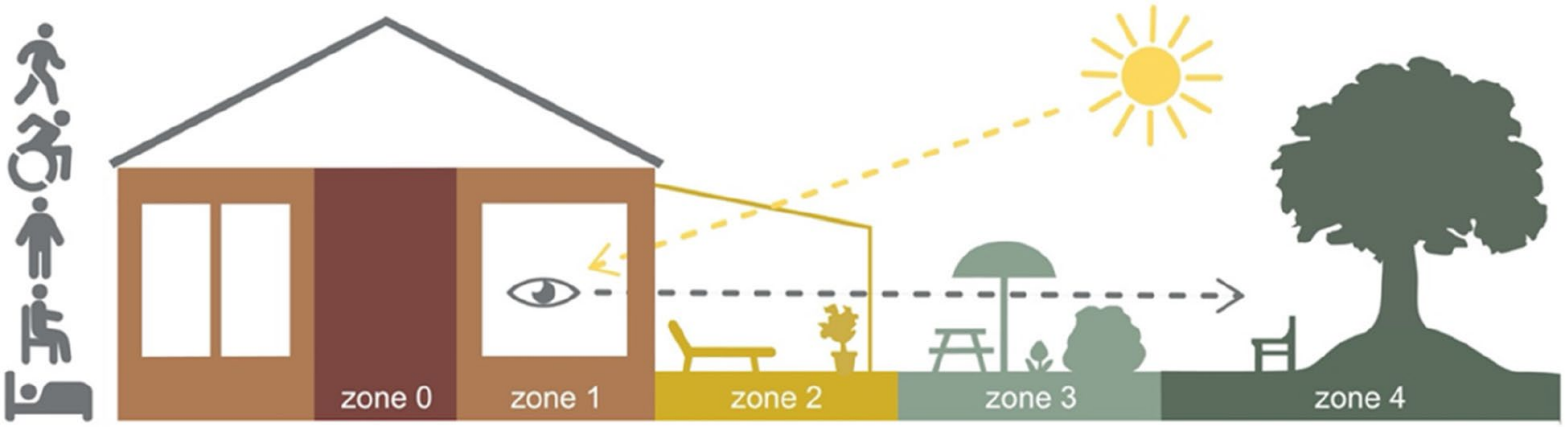
The zone model. Original illustration by Anna Bengtsson [4], reproduced with permission

To analyse how architectural conditions influence outdoor access, this study applies the zone model, which describes different levels of contact with outdoor environments [4]. Zone 1 refers to views through windows, Zone 2 to transitional environments between indoors and outdoors (such as entrances, balconies, and patios), Zone 3 to gardens, and Zone 4 to the surrounding neighbourhood environment. The present study focuses on Zones 1–3 and the transition points connecting them, as these represent environmental conditions directly related to the architectural design of RCFs. Zone 4 is excluded because access to neighbourhood environments involves additional aspects, including urban infrastructure, transportation, and community resources, which extend beyond the scope of this study. Within the zone model, transition points are understood as spatial elements that influence movement between zones and thereby shape opportunities for outdoor participation. The zone model also acknowledges that interaction with outdoor environments varies according to functional capacity, mobility, and cognitive status. Body positions include being in motion (walking or moving in a wheelchair), standing, sitting, and lying down [6], while cognitive status may range from high to low [36]. These variations influence how older adults experience and use different zones. Care workers are also included into the model, recognising that outdoor environments must support not only older adults’ needs but also the work practices required to provide personalised support [24] (Fig. 1).

## Methods

### Aim of the study

The aim of this study was to examine how storey configuration (single- versus multi-storey) and number of transition points were associated with opportunities for immediate access to outdoor environments within and adjacent to RCFs.

### Study design

A cross-disciplinary multi-methods design was employed [3], integrating three complementary datasets: national mapping ([28] Submitted to journal), transition points [24], and walking interviews [21]. The separate datasets were originally collected according to their specific aims. All studies had obtained ethical approvals. In the present study, the datasets were re-analysed and synthesised to examine how storey configuration and number of transition points shape opportunities for immediate access to outdoor environments in RCFs. The quantitative and qualitative components were analysed separately and integrated during interpretation. A national mapping of storey configuration (number of floors) in Swedish RCFs provided an overview of current conditions, while mapping of transition points between indoor and outdoor environments (from apartments to gardens) identified spatial variations related to storey configuration. Together, these components describe the structuring of the physical environment and how access between indoor and outdoor environments is organised. These structural environmental analyses provided a contextual basis for the re-analysis of walking interviews with older adults and care workers, which were originally conducted to explore experiences of environmental accessibility, mobility, and movement between indoor and outdoor environments.

### Study settings and sampling

**Fig. 2:**
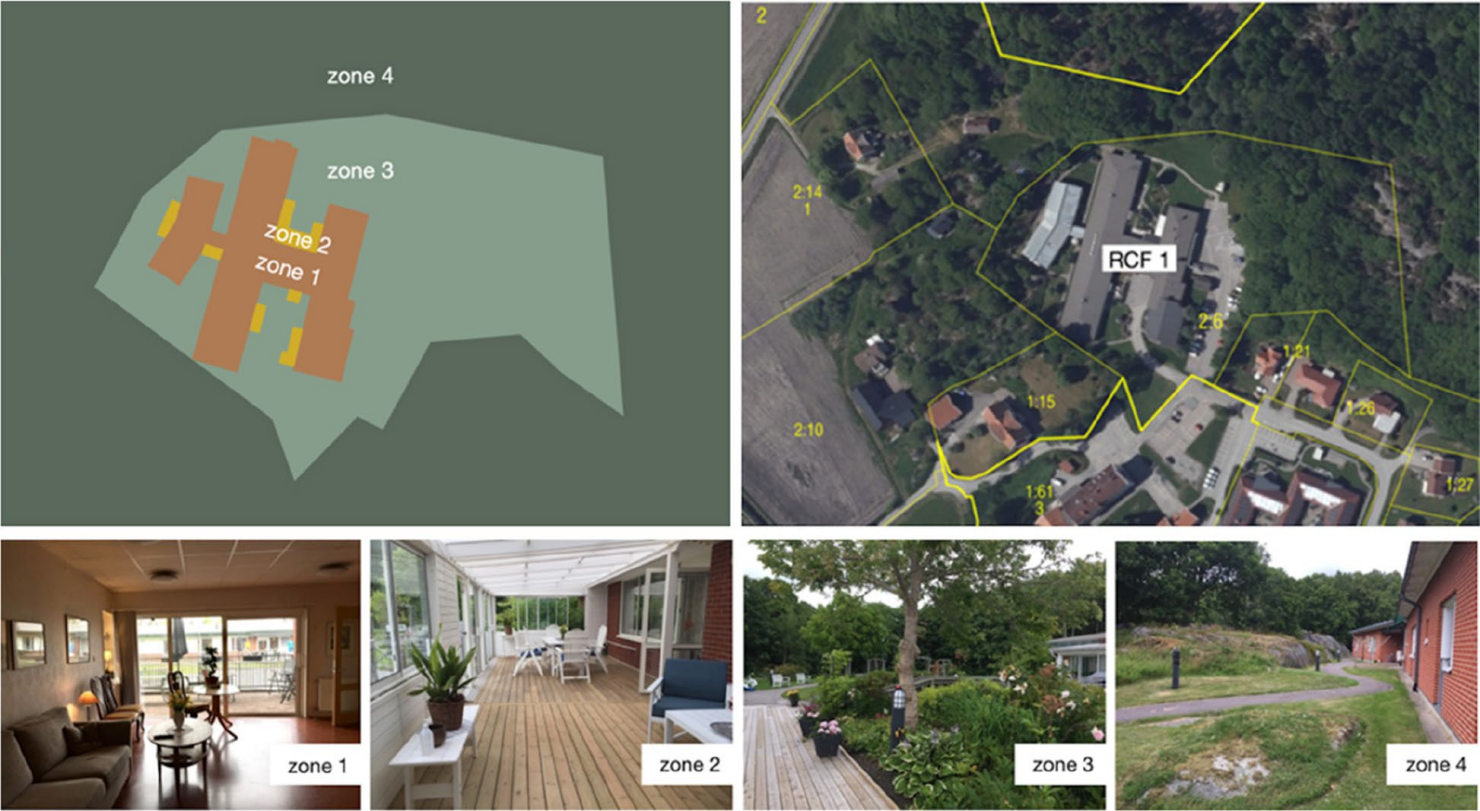
RCF 1 illustrated according to the zone model (top left), aerial view with marked plot boundaries (top right), and photographs of Zones 1–4. Illustrations and photographs by M. Liljegren. Reproduced from Liljegren [24] with permission

**Fig. 3:**
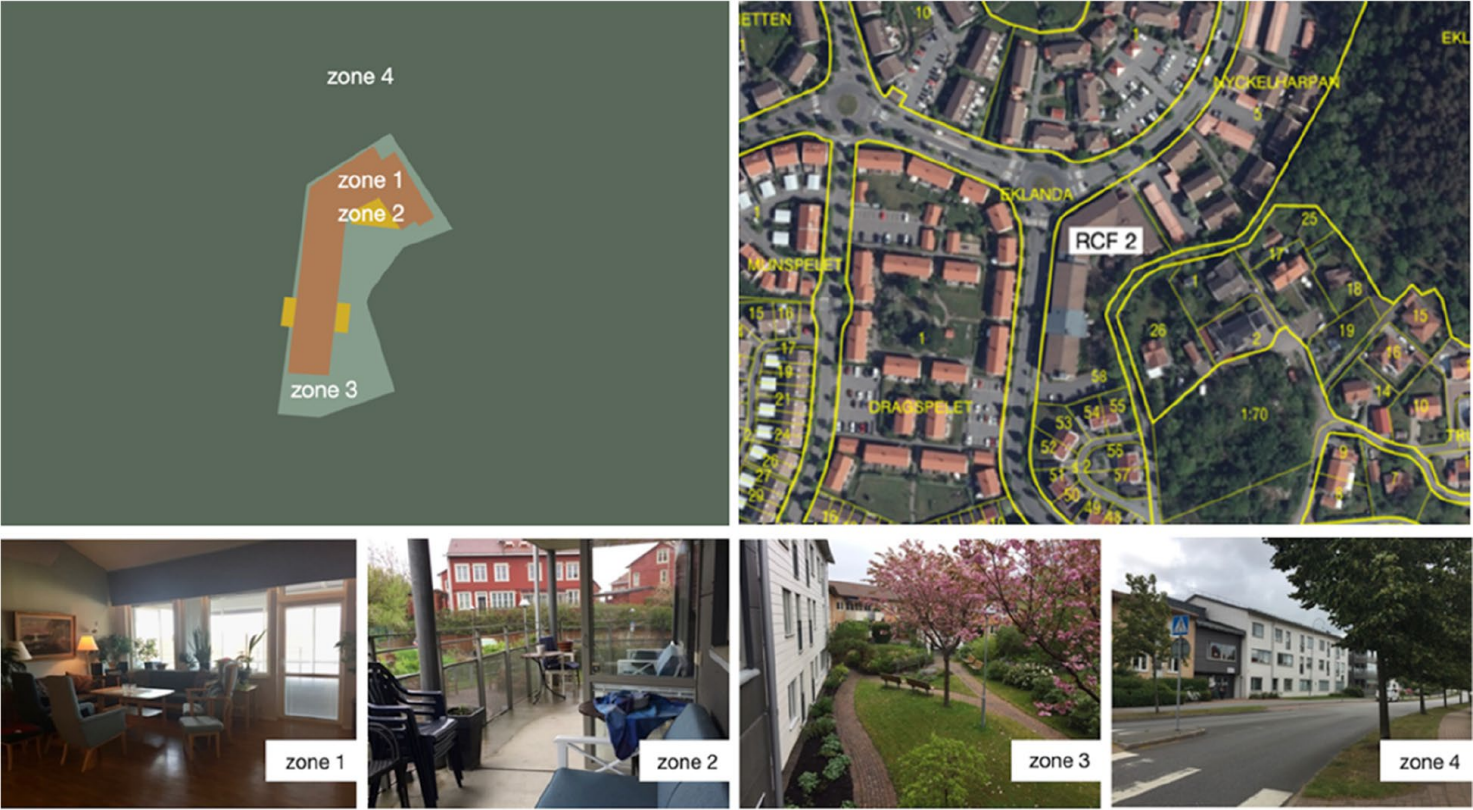
RCF 2 illustrated according to the zone model (top left), aerial view with marked plot boundaries (top right), and photographs of Zones 1–4. Illustrations and photographs by M. Liljegren. Reproduced from Liljegren [24] with permission

**Fig. 4:**
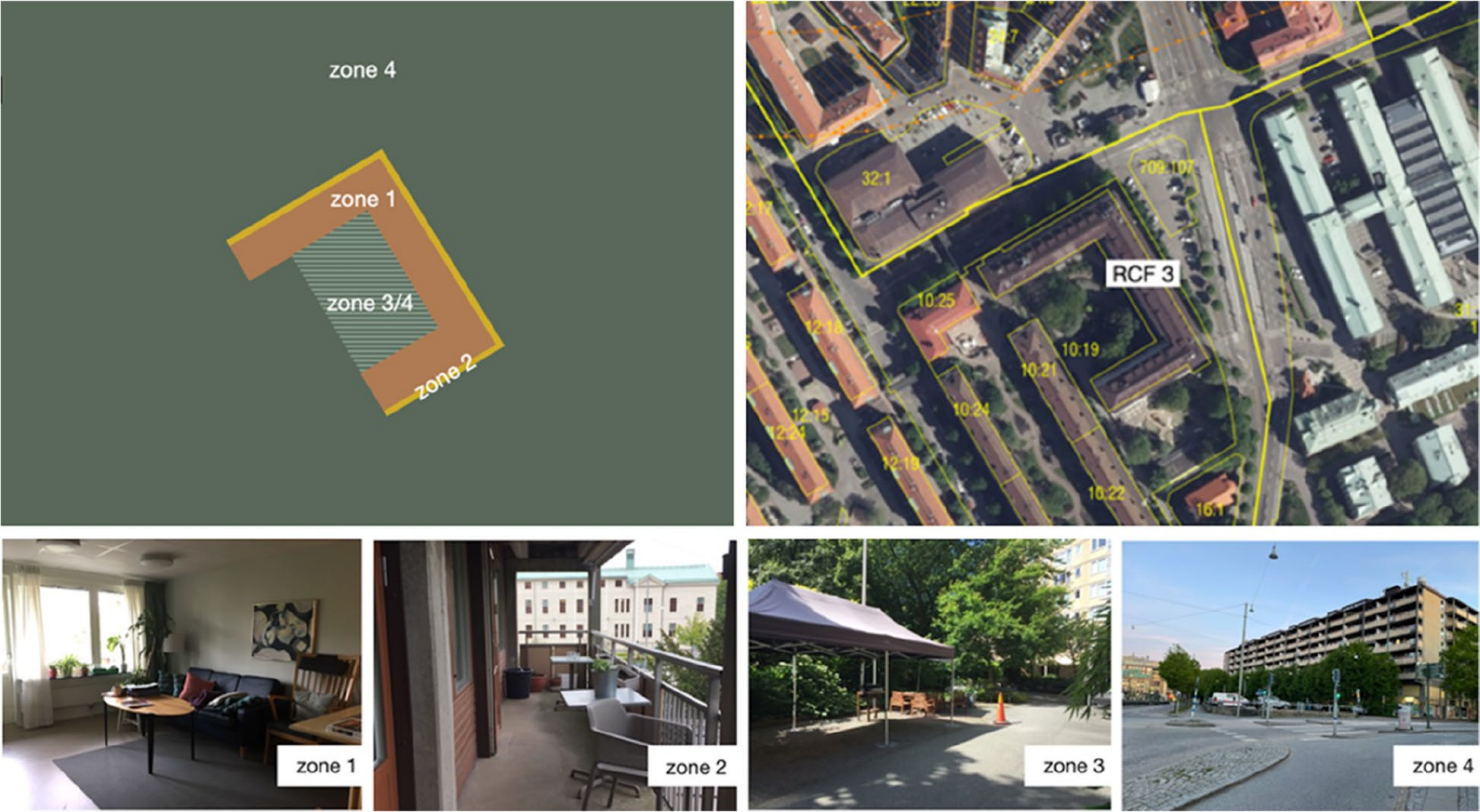
RCF 3 illustrated according to the zone model (top left), aerial view with marked plot boundaries (top right), and photographs of Zones 1–4. Illustrations and photographs by M. Liljegren. Reproduced from Liljegren [24] with permission

The number of transition points between indoor and outdoor environments were mapped and walking interviews conducted across three RCFs in the Gothenburg region, selected through strategic sampling based on a classification system developed by the Swedish Association of Local Authorities and Regions [43]. The RCFs were chosen to represent the three municipal groups (Group 1: large cities and municipalities close to large cities, Group 2: larger cities and in municipalities near larger cities, and Group 3: smaller cities/towns and rural municipalities) and to vary in number of floors (from one to nine). RCF 1 comprised a single floor with direct access to a garden surrounding the entire building. RCF 2 consisted of three floors with access to a backyard garden, while RCF 3 comprised nine floors and shared a park-like garden with other organisations. Figures 2, 3, 4 illustrate the three RCFs according to the zone model, including aerial views with plot boundaries and photographs of Zones 1–4. All photographs included in Figs. 2, 3, 4 were taken by the authors during data collection. No persons are visible in the photographs.

### Participants

The participants included both older adults and care workers recruited from RCFs 1–3. Purposive sampling was used to achieve variation in age, body position/functional capacity, years of residence, residential floor, and professional background. The older adult group comprised twelve older adults (nine women and three men). Four older adults were invited from each RCF, selected from a total population of 194 older adults. Written information about the study was provided to all older adults, and those interested in participating informed the managers, who proposed potential participants based on the inclusion criteria and desired variation. Final inclusion was determined by the researchers in connection with the interview based on willingness to participate and current health status. Inclusion criteria were the ability to communicate verbally in Swedish and the ability to participate in a walking interview. A diagnosis of severe cognitive decline was an exclusion criterion. Cognitive status was therefore considered through existing knowledge of severe cognitive impairment rather than assessed using a specific cognitive screening instrument. Several participants used mobility aids, such as walkers or wheelchairs. A detailed overview of participant characteristics is provided in Table 1.

**Table 1:** Description of older adults

Age	Body positions/functional capacity	Years living in RCFs	Residential floors
71–80 years old (*n* = 2)	In motion/standing independently (*n* = 2)	Less than one year (*n* = 2)	Floor 1 (*n* = 4)
81–90 years old (*n* = 4)	In motion/standing, used walker independently (*n* = 3)	One year (*n* = 3)	Floor 2 (*n* = 2)
91 years old or older (*n* = 6)	In motion/sitting, used wheelchair independently (*n* = 3)	Two years (*n* = 2)	Floor 3 (*n* = 3)
	In motion/sitting, used wheelchair, dependent on personal support (*n* = 4)	More than two years (*n* = 5)	Floor 4 (*n* = 1)
			Floor 5 (*n* = 1)
			Floor 6 (*n* = 1)

Care workers were recruited through managers at the participating RCFs. Managers informed eligible care workers about the study, and those interested in participating expressed their interest to the managers. Purposive sampling was used to ensure variation in professional role (social care and healthcare workers), years of experience in RCFs, age, and gender. The care worker group comprised eleven participants (nine women and two men) with diverse professional backgrounds. Participants were required to communicate verbally in Swedish. Details are provided in Table 2.

**Table 2:** Description of care workers

Profession	Years working in RCF	Age	Gender
Social care worker: Activity leader (*n* = 1)	Less than 1 year (*n* = 3)	21–30 years (*n* = 1)	Female (*n* = 9)
Social care worker: Assistant nurse (*n* = 2)	1–3 years (*n* = 3)	31–40 years (*n* = 2)	Male (*n* = 2)
Social care worker: Activity leader and assistant nurse (*n* = 1)	4–6 years (*n* = 1)	41–50 years (*n* = 5)	
Healthcare worker: Nurse (*n* = 3)	More than 6 years (*n* = 4)	51–60 years (*n* = 3)	
Healthcare worker: Occupational therapist (*n* = 2)			
Healthcare worker: Physiotherapist (*n* = 2)			

### Data collection

#### National mapping of storey configuration

Storey configuration in RCFs was mapped as part of a national study mapping access to Zones 1–4 across 2,036 RCFs in Sweden ([28] Submitted to journal), which was preceded by a study that developed a matrix and manual for the mapping [27]. Architectural drawings obtained from the building permit offices of the country’s 290 municipalities were used to calculate the number of floors at each RCF. Complete architectural drawings were obtained for 1,603 RCFs, enabling an analysis of the distribution between single- and multi-storey RCFs. The data were compiled in Microsoft Excel, and the extraction period spanned from 2022 to 2024.

#### Mapping of transition points between environments

The mapping of numbers of transition points was carried out through site visits to the respective RCFs and identified by walking from an apartment (Zone 1) to the garden (Zone 3). In RCF 1, the mapping was conducted on the first floor; in RCF 2, on the third floor; and in RCF 3, on the sixth floor. The selection of floor levels was based on where the participating older adults lived and where care workers included in the study were working. Because transition points were mapped on selected floors rather than throughout entire buildings, the analysis focused on illustrating how storey configuration influenced accessibility pathways. Comparisons were therefore based on identified spatial arrangements rather than complete counts of all possible transitions.

#### Walking interviews

Walking interviews were conducted individually with older adults and in focus groups with care workers. The interview data have previously been reported in separate studies [25, 26] and are re-analysed in the present study to address a different research aim by integrating them with national mapping and transition point analyses.

The semi-structured walking interviews with older adults were conducted in summer 2022 and followed an interview guide based on the zone model. Participants were invited to select and guide the interviewer to supportive or hindering locations within Zones 1–3. Discussions concerning Zone 4 were conducted in Zone 3 due to longer distances, participants’ varying physical capacity. The interviews lasted between 28 and 80 min, were audio-recorded, and transcribed verbatim.

Focus group interviews with care workers were also conducted in spring 2022 and followed an interview guide based on the zone model, focusing on experiences of the different zones and transitions between indoor and outdoor environments. The focus groups included visits to Zones 1–3 and discussions concerning Zone 4. Interviews lasted between 80 and 96 min, were audio-recorded, and transcribed verbatim.

Individual interviews with older adults enabled focused attention and adaptation to individual needs, whereas focus groups with care workers allowed participants to reflect on and expand each other’s experiences, facilitating discussion of shared and previously unarticulated perspectives [17]. During the walking interviews, the researchers documented observations related to environmental features and transitions between zones. The field notes were not analysed as a separate dataset but were used to contextualise and support interpretation of interview accounts.

### Data analysis

The mappings of storey configuration and number of transition points were analysed using descriptive statistics [37] and organised in Microsoft Excel. Storey configuration was summarised through distributions based on the number of floors. For transition points, each RCF was systematically reviewed to identify the points required to move from apartments to gardens. These steps were quantified to enable comparisons between the RCFs with different storey configuration.

The walking interviews with older adults and care workers were previously analysed in separate studies [25, 26]. In the present study, the interview data were re-analysed using qualitative content analysis [29] in relation to the current study aim, focusing on experiences of storey configuration, transition points, and how spatial conditions shaped opportunities for outdoor access. Relevant categories and findings were revisited and integrated with the analyses of architectural conditions.

The original qualitative analysis was conducted collaboratively within the research team, with initial coding performed by the first author and subsequent discussions to refine interpretations, resolve differences, and reach consensus regarding categories. Field notes describing environmental features and transitions were used to contextualise interview accounts. The qualitative findings were then integrated with the quantitative mapping of storey configuration and transition points to examine how spatial organisation may support or hinder outdoor access.

Organisational conditions were not examined systematically through analyses of policy documents, organisational guidelines, staffing data, care routines, or systematic observations. Instead, organisational aspects were reflected in participants’ accounts and considered as contextual aspects when interpreting opportunities for outdoor access. The results are presented in three sections corresponding to the study’s complementary data sources and interpreted through the lens of the zone model and storey configuration in RCFs. Participant quotations were used to support the findings and were abridged, edited for clarity, and translated into English.

## Results

First, results from the national mapping of the storey configuration in RCFs are reported. Second, results from the mapping of transition points between indoor and outdoor environments are presented as movement pathways between Zone 1 and Zone 3. Finally, results from the walking interviews with older adults and care workers are described, highlighting their experiences of how storey configuration shapes access to outdoor environments.

### National distribution of single- and multi-storey RCFs

In Sweden, 18.2% of RCFs are single-storey buildings, meaning that 81.8% comprise two or more floors. Two-storey buildings are the most common (32.6%), followed by three-storey buildings (22.6%) (Table 3). An analysis of single-storey RCFs stratified by the Swedish municipal group classification (Groups 1–3) reveals a clear geographical pattern. The proportion of single-storey RCFs increases with greater distance from big cities. In municipalities classified as “large cities and municipalities close to large cities” (Group 1), 8.7% of RCFs are single-storey. Correspondingly, 18.4% of RCFs in “larger cities and municipalities close to larger cities” (Group 2) are single-storey, while the highest proportion is found in “smaller cities/towns and rural municipalities” (Group 3), where 24.8% of RCFs have one floor.

**Table 3:** Number of floors at Swedish RCFs

Floor	Percent
1	18.2%
2	32.6%
3	22.6%
4	13.1%
5	6.4%
6	3.5%
7	1.6%
8	1.3%
9	0.4%
10	0.2%
11	0.1%
In total:	100.0% (*n* = 1,603)

### Number of transitions between indoor and outdoor environments

**Fig. 5:**
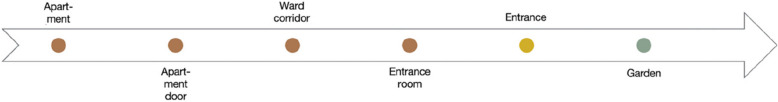
The six transition points from apartment to garden in RCF 1 (single-storey building). Illustration: M. Liljegren. Reproduced from Liljegren [24] with permission

**Fig. 6:**
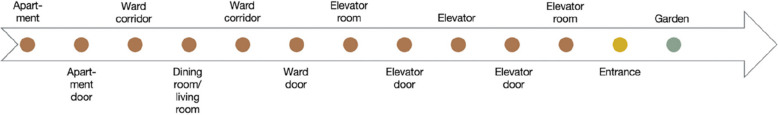
The 13 transition points from apartment to garden in RCF 2 (three-storey building). Illustration: M. Liljegren. Reproduced from Liljegren [24] with permission

**Fig. 7:**
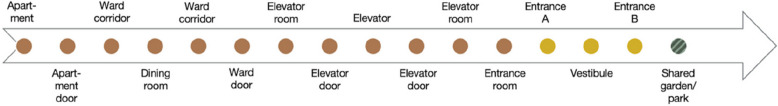
The 16 transition points from apartment to shared garden/park in RCF 3 (nine-storey building). Illustration: M. Liljegren. Reproduced from Liljegren [24] with permission

The results indicate a pattern in which the storey configuration of the three included RCFs is associated with the number of transitions required to move from apartments (Zone 1) to the garden (Zone 3). Across the three RCFs, buildings with more storeys were characterised by a greater number of transition points. In RCF 1 (single-storey building), six transition points were identified between apartments and the garden (Fig. 5). In RCF 2 (three-storey building), 13 transition points were identified (Fig. 6). The highest number was observed in RCF 3 (nine-storey building), where 16 transition points were identified (Fig. 7). These findings suggest that, in the included RCFs, multi-storey RCFs may involve more complex Zone 1–Zone 3 pathways.

### Experiences of single- versus multi-storey RCFs

Four categories were identified, reflecting older adults’ and care workers’ experiences of outdoor access in single- and multi-storey RCFs.

#### Older adults’ experiences of single-storey RCFs

This category describes older adults’ experiences of living in an RCF located at ground level. The category encompasses two subcategories: *The importance of proximity* and *Dependence on support during indoor–outdoor transitions*.

*The importance of proximity:* Older adults described proximity between indoor and outdoor environments as important for everyday access to outdoor environments. In the single-storey RCF, reaching the garden often involved passing through only one door from shared indoor environments, providing a direct route outdoors.

*Dependence on support during indoor–outdoor transitions:* Despite the direct connection between indoor and outdoor environments, some older adults described needing support from care workers during transitions due to functional limitations. One older adult using a wheelchair explained: “As long as the doors can be opened (via automatic push-button door openers, no support from care workers is needed to go outside).” (Older man, aged 81–90, using a powered wheelchair). However, minor environmental features, such as thresholds, could influence independence. Another older adult described needing personal support: “To get that (the wheelchair) over the threshold so I don’t have to bother others every time.” (Older woman, aged > 91 years or older, using wheelchair).

#### Care workers’ experiences of single-storey RCFs

This category describes care workers’ experiences of working in an RCF located at ground level. The category encompasses two subcategories: *Easy access to outdoor environments* and *Ability to maintain overview*.

*Easy access to outdoor environments:* Care workers described single-storey RCFs as providing easy access to outdoor environments. The absence of stairs and elevators was described as facilitating access to patios and gardens for both older adults and care workers. Care workers described short distances between indoor and outdoor environments as enabling frequent use of patios and gardens in everyday care and rehabilitation activities. Wide openings, minimal thresholds, and smooth transitions were described as facilitating movement between the environments. One care worker described the connection between a conservatory and the patio: “It is a large door and you can open it up, and it is easy to roll out onto the deck.” (Social care worker). The same care worker emphasized the importance of avoiding physical barriers: “That there are not a lot of edges and that it is not cramped… it makes a big difference.” (Social care worker). Care workers described that accessible outdoor environments made it possible to include shorter outdoor activities, such as brief walks, as part of everyday care and rehabilitation.

*Ability to maintain overview:* Care workers described that the single-storey layout enabled visual oversight of older adults across indoor and outdoor environments. They reported being able to support older adults outdoors while maintaining visual contact with older adults remaining indoors through windows. Similarly, older adults spending time outdoors could be observed from inside the building. Care workers contrasted this with situations where older adults were outdoors while care workers were located on another floor, describing that the single-storey layout enabled continued overview of activities in different environments.

#### Older adults’ experiences of multi-storey RCFs

This category describes older adults’ experiences of living in a multi-storey RCF. The category encompasses four subcategories: *Limited or absent outdoor stays, Dependence on care workers, The elevator as an enabler of movement between storeys*, and *Discomfort and limitations associated with elevator use*.

*Limited or absent outdoor stays:* Outdoor stays were described as limited or absent for several older adults. Although gardens were available, they were not always used as part of everyday life. Some older adults described visiting outdoor environments infrequently, while others had never accessed the garden. One older adult stated: “I haven’t even been on the back side here.” (Older woman, aged > 91 years or older, using wheelchair). Some older adults also expressed uncertainty about what the garden offered and described that outdoor areas were not part of their everyday activities. For older adults using mobility aids, outdoor movement was affected by the conditions of outdoor routes. Level differences and uneven transitions along outdoor routes were described as creating difficulties during movement with mobility aids. One older adult using a wheelchair described the physical impact of passing over such surfaces: “It becomes difficult. You feel shocks throughout the whole body.” (Older woman, 71–80 years old, using wheelchair).

*Dependence on care workers:* Outdoor activities were described as dependent on support from care workers when older adults could not manage mobility independently. Support was needed for moving within the building and between indoor and outdoor environments. One older adult stated: “When I get help with it (outdoor stays), then I can do it. But I mean, I can’t do it myself.” (Older woman, 71–80 years old, using wheelchair). The same older adult described the difficulty of repeatedly requesting support: “It is difficult to get help, constantly asking for help and help and help and help.” (Older woman, 71–80 years old, using wheelchair). Environmental features, including doors and thresholds, were described as affecting the need for support during transitions. One older adult described being able to leave the building but having difficulties returning: “Yes, I can get out… it is worse getting back in. Then I have to call for assistance.” (Older man, aged 81–90, using a powered wheelchair).

*The elevator as an enabler of movement between storeys:* The elevator was described as enabling movement between storeys and outdoor environments. For some older adults, it supported independent movement within the building. One older adult using a wheelchair stated: “It is just a matter of pressing a button.” (Older man, aged > 91 years or older, using wheelchair).The design of the elevator space influenced how easily it could be used. One older adult described preferring a larger elevator because it allowed turning with a wheelchair, whereas “the other one (another elevator) is a bit narrow.” (Older man, aged > 91 years or older, using wheelchair).

*Discomfort and limitations associated with elevator use:* Elevator use was described as uncomfortable by some older adults. Elevators without windows were experienced as unpleasant and could cause nausea. One older adult using a walker stated: “When I ride the elevator, it feels kind of… oh, how disgusting (the elevator moves). I think it’s disgusting… It makes me a little nauseous.” (Older woman, > 91 years old or older, using walker). Some older adults using mobility aids also described uncertainty when entering and exiting elevators. These situations could make elevator use difficult and could require support from care workers.

#### Care workers’ experiences of multi-storey RCFs

This category describes care workers’ experiences of working in a multi-storey RCF. The category encompasses three subcategories: *Spatial inequalities affecting outdoor access, The elevator as both enabler and constraint,* and *Organisational conditions influencing outdoor care and rehabilitation.*

*Spatial inequalities affecting outdoor access:* Care workers described that opportunities for outdoor access differed depending on the location of residential units within the building. Older adults living on ground floors were described as having more direct access to patios and gardens, whereas those living on upper floors often faced longer and more complex routes. Care workers described that reaching outdoor environments from upper floors could involve travelling through long corridors, using elevators, and navigating several transitions before reaching the exit. One care worker explained: “It becomes three steps to get outside when you are up here. They (older adults) have to roll along a 16-m corridor, use several buttons, go all the way down, and then out through an exterior door.” (Social care worker). Care workers also described that the physical distance between residential areas and outdoor environments could limit older adults’ ability to access the outdoors. Some older adults were unable to manage the distance independently, and the effort required to reach outdoor environments was described as a barrier. Care workers contrasted multi-storey RCFs with single-storey RCFs, where access to outdoor environments was perceived as easier and outdoor activities could occur more spontaneously. One care worker stated: “The big advantage is the accessibility somehow, that it is on one floor and that it is easy to get outside.” (Nurse).

*The elevator as both enabler and constraint:* Care workers described the elevator as an important prerequisite for enabling movement between residential floors and outdoor environments. At the same time, elevator use was described as adding complexity to outdoor access by requiring additional time, coordination, and assistance. Care workers described that the need to wait for, operate, and travel by elevator could make going outdoors a more demanding activity.

*Organisational conditions influencing outdoor care and rehabilitation:* Care workers described that opportunities to provide outdoor care and rehabilitation were influenced by everyday organisational conditions. Aspects mentioned included staffing levels, available time, communication, team planning, and care workers’ engagement. Care workers described that outdoor activities required initiative and coordination within the team, and that opportunities were dependent on whether care workers prioritised and facilitated such activities. One care worker explained: “I think the conditions are there. A lot is also about the commitment of those of us who work here and time and that bit. There may often be time, but you have to … there has to be a little will, too, in some way. It could also be what is missing.” (Nurse).

## Discussion

### Storey configuration and transition points as conditions for outdoor access

Interpreted through the perspectives of age-friendly environments [51, 52], person-centred practice [31, 32], and the zone model [4, 24, 36], the findings demonstrate that storey configuration and number of transition points are structural conditions shaping opportunities for outdoor access. By combining national mapping, transition point analyses, and lived experiences, this study extends previous research by showing that outdoor access depends not only on the availability and quality of outdoor environments, but also on the architectural pathways required to reach them.

Previous research has largely focused on neighbourhood accessibility, access to outdoor environments, and green space qualities [10, 27, 40]. The present findings shift attention to the internal organisation of RCFs, demonstrating that access is generated through the interaction between storey configuration, number of transition points, and possibilities for everyday use. From an age-friendly environments perspective, outdoor environments cannot be considered accessible simply because they exist, they must also be practically reachable for older adults with diverse functional and cognitive abilities.

The national mapping showed that most Swedish RCFs are multi-storey buildings, indicating that vertical layouts represent the dominant residential care context. Across the three included RCFs, a pattern was observed in which buildings with more storeys also had more complex pathways between Zone 1 and Zone 3. However, accessibility was not determined by storey configuration alone. Rather, the number of transition points shaped the effort required to reach outdoor environments. Thus, buildings with similar numbers of storeys may provide different opportunities for outdoor participation depending on how these transitions are designed and organised.

### Environmental design and outdoor person-centred care and rehabilitation

The walking interviews [21] further demonstrated that storey configuration influenced not only physical access but also how outdoor activities could be integrated into everyday care and rehabilitation practices. In the single-storey RCF, shorter distances and fewer transition points enabled more spontaneous use of outdoor environments. Care workers also described improved visual oversight between indoor and outdoor environments, supporting supervision while maintaining opportunities for outdoor activities. In contrast, multi-storey RCFs involved longer and more complex movement pathways, making outdoor activities more dependent on planning, coordination, and support.

Although organisational conditions were not the primary focus of this study, care workers consistently identified staffing, time, communication, planning, and engagement as aspects influencing outdoor participation. These findings align with previous research demonstrating the importance of organisational routines for outdoor use in RCFs [5, 25]. Leadership has also been identified as an important organisational condition for supporting the use of outdoor environments in RCFs [14]. The present study extends this knowledge by showing that organisational aspects operate within architectural structures that may either support or hinder outdoor person-centred care and rehabilitation.

The findings also highlight that seemingly minor environmental features, such as thresholds, doors, and elevators, influenced whether older adults could move independently or required support. These experiences indicate that transition points are not only physical barriers but may also shape experiences of insecurity and a diminished sense of control during movement. Independence therefore emerged as a relational phenomenon shaped by the interaction between individual abilities and environmental demands. Rather than functioning as isolated barriers, transition points accumulated along movement pathways, increasing the practical effort required to access outdoor environments. As the number of transition points increased, opportunities for spontaneous outdoor activities became increasingly dependent on care worker availability and organisational resources. Together, these findings position storey configuration and number of transition points as important architectural conditions influencing the implementation of outdoor person-centred care and rehabilitation.

### Implications for age-friendly RCF design, planning, and practice

The findings have implications for the design, planning, and organisation of age-friendly RCFs. Equitable access to outdoor environments depends not only on providing gardens, patios, or other outdoor environments, but also on how movement between residential areas and these environments is organised. Consequently, older adults within the same RCF may experience different opportunities for outdoor participation depending on the location of their apartment and the complexity of the route required to reach outdoor environments.

The findings suggest that number of transition points should be considered alongside conventional accessibility requirements when designing new RCFs and evaluating existing facilities. Although accessibility standards often address individual features, such as door widths, thresholds, and elevators, this study highlights the importance of considering the cumulative effect of multiple transitions along everyday movement routes. Shorter and more direct connections between residential units and outdoor environments and reduced unnecessary transitions may support greater independence and participation. For multi-storey RCFs, direct elevator access from residential areas to gardens could be considered to reduce transition points and create more direct routes to outdoor environments.

For existing multi-storey RCFs, adaptations such as automatic door openers and reduced thresholds may help decrease environmental barriers. However, the findings also demonstrate that physical accessibility alone does not determine outdoor participation. Care workers described outdoor activities as dependent on organisational resources and everyday priorities. Architectural and organisational conditions should therefore be understood as interacting determinants of outdoor access [9, 24].

Although conducted in Sweden, the findings have relevance for residential care systems internationally. As many countries respond to population ageing by developing higher-density RCFs while promoting person-centred care and rehabilitation, and age-friendly principles, greater attention is needed to the internal spatial organisation of RCFs [52]. Decisions regarding storey configuration and transition pathways may influence opportunities for outdoor participation throughout older adults’ daily lives.

### Methodological considerations

A major strength of this study is the integration of three complementary data sources: national mapping of storey configuration, transition point analyses, and walking interviews [21] with older adults and care workers. This multi-methods approach [3] enabled examination of outdoor access across structural, environmental, and experiential levels, strengthening the understanding of how architectural conditions shape opportunities for outdoor person-centred care and rehabilitation.

Several limitations should be considered. The transition point analysis was conducted in three purposively selected RCFs and focused on one storey in each building. While this enabled systematic comparisons, analyses of a larger number of RCFs are needed to draw more robust conclusions about the relationship between storey configuration and the number of transition points. Although this enabled systematic comparisons, it did not capture variations in travel distance, time, or physical effort across different parts of the RCFs. The qualitative findings were based on a small purposive sample from three RCFs and should therefore be interpreted as analytically transferable rather than statistically generalisable. Because recruitment of older adults was facilitated through RCF managers, participants may represent care workers who were more willing or able to engage in research activities. Participation requirements may also have limited the inclusion of older adults with severe cognitive impairment or very limited mobility, groups whose experiences of environmental barriers require further attention. In addition, the study focused primarily on outdoor access within the RCF (Zones 1–3), rather than neighbourhood accessibility more broadly. Despite these limitations, the combination of spatial analyses and lived experiences provides insights into how architectural configuration influences outdoor access. A further limitation is that organisational conditions were not examined systematically and were considered only as contextual aspects based on participants' accounts. Future research should integrate architectural and organisational perspectives across a wider range of RCFs to better understand how built environment design and organisational conditions interact to support outdoor person-centred care and rehabilitation.

## Conclusion

This study shows that storey configuration and number of transition points shape access to outdoor environments in RCFs and thereby conditions for outdoor person-centred care and rehabilitation. Single-storey RCFs were characterised by more direct pathways to outdoor environments, whereas the included multi-storey RCFs had a greater number of transition points between indoor and outdoor environments. Older adults described these more complex pathways as requiring greater support from care workers and organisational resources. These findings highlight storey configuration and transition points as structural conditions influencing outdoor access in RCFs, with implications also for age-friendly design and planning of RCFs.

## Data Availability

No datasets were generated or analysed during the current study.

## Abbreviation

RCF: Residential care facility
